# Clinicopathological features and prognosis of primary small bowel adenocarcinoma: a large multicenter analysis of the JSCCR database in Japan

**DOI:** 10.1007/s00535-024-02081-3

**Published:** 2024-02-27

**Authors:** Ken Yamashita, Shiro Oka, Takeshi Yamada, Keigo Mitsui, Hironori Yamamoto, Keiichi Takahashi, Akio Shiomi, Kinichi Hotta, Yoji Takeuchi, Toshio Kuwai, Fumio Ishida, Shin-Ei Kudo, Shoichi Saito, Masashi Ueno, Eiji Sunami, Tomoki Yamano, Michio Itabashi, Kazuo Ohtsuka, Yusuke Kinugasa, Takayuki Matsumoto, Tamotsu Sugai, Toshio Uraoka, Koichi Kurahara, Shigeki Yamaguchi, Tomohiro Kato, Masazumi Okajima, Hiroshi Kashida, Yoshito Akagi, Hiroaki Ikematsu, Masaaki Ito, Motohiro Esaki, Masaya Kawai, Takashi Yao, Madoka Hamada, Takahiro Horimatsu, Keiji Koda, Yasumori Fukai, Koji Komori, Yusuke Saitoh, Yukihide Kanemitsu, Hiroyuki Takamaru, Kazutaka Yamada, Hiroaki Nozawa, Tetsuji Takayama, Kazutomo Togashi, Eiji Shinto, Takehiro Torisu, Akira Toyoshima, Naoki Ohmiya, Takeshi Kato, Eigo Otsuji, Shinji Nagata, Yojiro Hashiguchi, Kenichi Sugihara, Yoichi Ajioka, Shinji Tanaka

**Affiliations:** 1 Department of Gastroenterology, Hiroshima University Hospital, 1-2-3, Kasumi, Minami-Ku, Hiroshima, 734-8551 Japan; 2 Department of Gastrointestinal and Hepato-Biliary-Pancreatic Surgery, Nippon Medical School, Tokyo, Japan; 3 Department of Gastroenterology, Nippon Medical School, Graduate School of Medicine, Tokyo, Japan; 4 Department of Medicine, Division of Gastroenterology, Jichi Medical University, Tochigi, Japan; 5 Department of Colorectal Surgery, Tokyo Metropolitan Cancer and Infectious Diseases Center Komagome Hospital, Tokyo, Japan; 6 Division of Colon and Rectal Surgery, Shizuoka Cancer Center, Shizuoka, Japan; 7 Division of Endoscopy, Shizuoka Cancer Center, Shizuoka, Japan; 8 Department of Gastrointestinal Oncology, Osaka International Cancer Institute, Osaka, Japan; 9 Department of Gastroenterology, National Hospital Organization Kure Medical Center and Chugoku Cancer Center, Hiroshima, Japan; 10 Digestive Disease Center, Showa University Northern Yokohama Hospital, Kanagawa, Japan; 11 Department of Lower Gastrointestinal Medicine, Cancer Institute Hospital of the Japanese Foundation for Cancer Research, Tokyo, Japan; 12 Department of Gastroenterological Surgery, Toranomon Hospital, Tokyo, Japan; 13 Department of Surgery, Kyorin University School of Medicine, Tokyo, Japan; 14 Division of Lower Gastrointestinal Surgery, Department of Surgery, Hyogo College of Medicine, Hyogo, Japan; 15 Department of Surgery, Institute of Gastroenterology, Tokyo Women’s Medical University, Tokyo, Japan; 16 Department of Gastroenterology and Hepatology, Tokyo Medical and Dental University, Tokyo, Japan; 17 Department of Gastrointestinal Surgery, Tokyo Medical and Dental University, Tokyo, Japan; 18 Division of Gastroenterology, Department of Internal Medicine, Iwate Medical University, Iwate, Japan; 19 Department of Diagnostic Pathology, Iwate Medical University, Iwate, Japan; 20 Department of Gastroenterology and Hepatology, Gunma University Graduate School of Medicine, Gunma, Japan; 21 Division of Gastroenterology, Matsuyama Red Cross Hospital, Ehime, Japan; 22 Department of Gastroenterological Surgery, Saitama Medical University International Medical Center, Saitama, Japan; 23 Division of Gastroenterology and Hepatology, Department of Internal Medicine, The Jikei University School of Medicine, Tokyo, Japan; 24 Department of Surgery, Hiroshima City Hiroshima Citizens Hospital, Hiroshima, Japan; 25 Department of Gastroenterology and Hepatology, Kindai University Faculty of Medicine, Osaka, Japan; 26 Department of Surgery, Kurume University School of Medicine, Fukuoka, Japan; 27 Department of Gastroenterology and Endoscopy, National Cancer Center Hospital East, Chiba, Japan; 28 Department of Colorectal Surgery, National Cancer Center Hospital East, Chiba, Japan; 29 Division of Gastroenterology, Department of Internal Medicine, Faculty of Medicine, Saga University, Saga, Japan; 30 Department of Coloproctological Surgery, Faculty of Medicine, Juntendo University, Tokyo, Japan; 31 Department of Human Pathology, Juntendo University Graduate School of Medicine, Tokyo, Japan; 32 Department of Gastrointestinal Surgery, Kansai Medical University Hospital, Osaka, Japan; 33 Department of Clinical Oncology, Kyoto University Hospital, Kyoto, Japan; 34 Department of Surgery, Teikyo University Chiba Medical Center, Chiba, Japan; 35 Department of Gastroenterology, Maebashi Red Cross Hospital, Gunma, Japan; 36 Department of Gastroenterological Surgery, Aichi Cancer Center Hospital, Aichi, Japan; 37 Department of Gastroenterology, Asahikawa City Hospital, Asahikawa, Hokkaido Japan; 38 Department of Colorectal Surgery, National Cancer Center Hospital, Tokyo, Japan; 39 Endoscopy Division, National Cancer Center Hospital, Tokyo, Japan; 40 Department of Surgery, Coloproctology Center Takano Hospital, Kumamoto, Japan; 41 Department of Surgical Oncology, The University of Tokyo, Tokyo, Japan; 42 Department of Gastroenterology and Oncology, Tokushima University Graduate School of Biomedical Sciences, Tokushima, Japan; 43 Department of Coloproctology, Aizu Medical Center, Fukushima Medical University, Fukushima, Japan; 44 Department of Surgery, National Defense Medical College, Tokorozawa, Saitama Japan; 45 Department of Medicine and Clinical Science, Graduate School of Medical Sciences, Kyushu University, Fukuoka, Japan; 46 Department of Colorectal Surgery, Japanese Red Cross Medical Center, Tokyo, Japan; 47 Department of Advanced Endoscopy, Fujita Health University School of Medicine, Aichi, Japan; 48 Department of Surgery, National Hospital Organization Osaka National Hospital, Osaka, Japan; 49 Division of Digestive Surgery, Department of Surgery, Kyoto Prefectural University of Medicine, Kyoto, Japan; 50 Department of Gastroenterology, Hiroshima City North Medical Center Asa Citizens Hospital, Hiroshima, Japan; 51 Department of Surgery, Japanese Red Cross Omori Hospital, Tokyo, Japan; 52 Tokyo Medical and Dental University, Tokyo, Japan; 53 Division of Molecular and Diagnostic Pathology, Niigata University Graduate School of Medical and Dental Sciences, Niigata, Japan

**Keywords:** Primary small bowel adenocarcinoma, Capsule endoscopy, Double-balloon endoscopy, Lynch syndrome, Prognosis

## Abstract

**Background:**

The clinicopathological features and prognosis of primary small bowel adenocarcinoma (PSBA), excluding duodenal cancer, remain undetermined due to its rarity in Japan.

**Methods:**

We analyzed 354 patients with 358 PSBAs, between January 2008 and December 2017, at 44 institutions affiliated with the Japanese Society for Cancer of the Colon and Rectum.

**Results:**

The median age was 67 years (218 males, 61.6%). The average tumor size was 49.9 (7–100) mm. PSBA sites consisted of jejunum (66.2%) and ileum (30.4%). A total of 219 patients (61.9%) underwent diagnostic small bowel endoscopy, including single-balloon endoscopy, double-balloon endoscopy, and capsule endoscopy before treatment. Nineteen patients (5.4%) had Lynch syndrome, and 272 patients (76.8%) had symptoms at the initial diagnosis. The rates for stages 0, I, II, III, and IV were 5.4%, 2.5%, 27.1%, 26.0%, and 35.6%, respectively. The 5-year overall survival rates at each stage were 92.3%, 60.0%, 75.9%, 61.4%, and 25.5%, respectively, and the 5-year disease-specific survival (DSS) rates were 100%, 75.0%, 84.1%, 59.3%, and 25.6%, respectively. Patients with the PSBA located in the jejunum, with symptoms at the initial diagnosis or advanced clinical stage had a worse prognosis. However, multivariate analysis using Cox-hazard model revealed that clinical stage was the only significant predictor of DSS for patients with PSBA.

**Conclusions:**

Of the patients with PSBA, 76.8% had symptoms at the initial diagnosis, which were often detected at an advanced stage. Detection during the early stages of PSBA is important to ensure a good prognosis.

## Introduction

Primary small bowel cancer involving various histological tumors, such as adenocarcinoma, carcinoid, malignant lymphoma, gastrointestinal stromal tumor, and sarcoma, is relatively uncommon; however, the number of cases with this condition has increased in recent years [1–4]. According to the previous reports [5, 6], the rate of primary small bowel adenocarcinoma (PSBA) is < 3% of all gastrointestinal cancers. Furthermore, as more than half of all PSBAs occur in the duodenum, PSBAs of the jejunum and ileum are particularly rare [2–4].

Risk factors for PSBA include hereditary diseases, such as familial adenomatous polyposis, Lynch syndrome, and Peutz–Jeghers syndrome, and chronic inflammatory diseases, such as Crohn’s disease, celiac disease, and obesity [7–13]. Although hereditary and chronic inflammatory diseases are predisposing factors for PSBA in Western countries [8], a Japanese multicenter study reported that these factors were not associated with the development of PSBA [7]. The risk factors may differ among racial groups.

However, PSBA has many genetic alterations (*KRAS, TP53, APC, SMAD4, and PIK3CA*); the prevalence of *APC* mutations in PSBA was significantly lower than that in colorectal cancer, suggesting a distinct molecular background [8, 14, 15]. Approximately 20% of PSBA cases showed mismatch-repair deficiency [8, 15], which may have clinical relevance with the therapeutic indications for immune checkpoint inhibitors and the existence of Lynch syndrome.

Conventionally, it is difficult to diagnose PSBA using only external ultrasonography, small bowel radiography, or contrast-enhanced computed tomography; therefore, the diagnosis is often made using surgical resection [16, 17]. Recent advances in endoscopic procedures have led to the widespread use of capsule and balloon endoscopes, which have dramatically improved the diagnostic ability for small bowel diseases [18, 19]. However, PSBA is often diagnosed at an advanced stage owing to symptoms of stenosis, metastasis, or peritoneal dissemination [2–4, 7, 8], and the tumor stage is the most important prognostic factor in PSBA [16, 20]. Therefore, there is an urgent need to clarify the development and progression of PSBA and work toward its early detection. Other factors associated with poor prognosis in PSBA include poor differentiation, positive margins, duodenal location, lymphovascular invasion, lymph-node metastasis, carcinoembryonic antigen and carbohydrate antigen 19–9 levels, presence of symptoms at diagnosis, male sex, black ethnicity, and older age [1, 21]. In this study, we focused on the existence of symptoms at initial diagnosis and PSBA sites related to prognosis.

Currently, surgical resection is frequently performed for PSBA; however, standard surgical procedures, such as the extent of lymph-node dissection and length of bowel resection, have not been established. Therefore, in Japan, the extent of surgical resection is usually determined by clinical surgeons in accordance with the Japanese Classification of Colorectal, Appendiceal, and Anal Carcinoma (JCCAC) [22]. In this study, we analyzed the clinicopathological features and prognosis of PSBA using data from a large multicenter study, according to the JCCAC.

## Methods

**Fig. 1:**
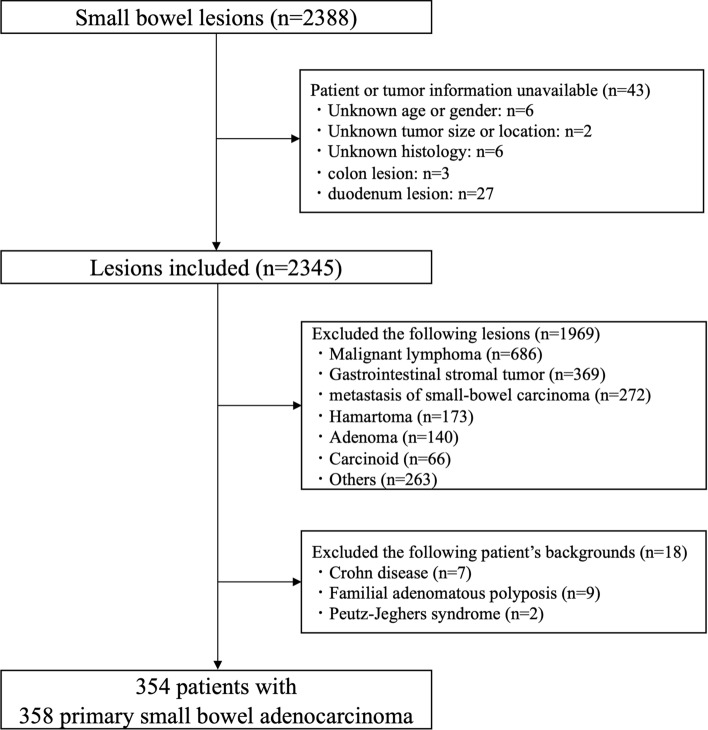
Flowchart for enrolled tumors and patients in this study

We collected data of 2388 primary small bowel lesions between January 2008 and December 2017 from 44 institutions affiliated with the Japanese Society for Cancer of the Colon and Rectum (JSCCR) in Japan. The JSCCR was established to conduct research on colorectal cancer to introduce measures that may improve its diagnosis and treatment. The 44 affiliated institutions included in this study are university hospitals, cancer centers, and major regional hospitals, and are all core Japanese colorectal cancer treatment hospitals. PSBA sites were limited to the jejunum and ileum (excluding the duodenum). A total of 2030 lesions were excluded for the following reasons: unavailable patient or tumor essential information, histology was not adenocarcinoma (malignant lymphoma, gastrointestinal stromal tumor, metastasis of PSBA, hamartoma, adenoma, or carcinoid), and presence of underlying conditions (Crohn’s disease, familial adenomatous polyposis, and Peutz–Jeghers syndrome). Compared to diagnosis of other diseases, it is more difficult to distinctly diagnose Lynch syndrome based on the data (clinical features, history of malignancy of other organs, and family history of malignancy) from the multiple institutions; therefore, it was not excluded from this study (Fig. 1). The pathological features and TNM classification of PSBA were evaluated according to the JCCAC eighth edition [22]. The JCCAC differs from the UICC-TNM classification [23] of PSBA in the T and N categories. The JCCAC T category is defined as follows: Tis, tumor is confined to the mucosa and does not invade the submucosa; T1, tumor is confined to the submucosa and does not invade the muscularis propria; T2, tumor invasion extends to, but not beyond, the muscularis propria; T3, tumor invasion beyond the muscularis propria; and T4, tumor invades or perforates the serosa or directly invades other organs or structures (at sites with serosa, the tumor grows into the subserosa, and at sites with no serosa, the tumor grows into the adventitia). Similarly, the N category is defined as follows: N1, metastasis in 1–3 pericolic/perirectal or intermediate lymph nodes; N2, metastasis in four or more pericolic/perirectal or intermediate lymph nodes; and N3, metastasis in the main lymph node(s).

The patient data were approved by the ethics committee of the JSCCR (approval date: August 30, 2019) and each participating institution. The study was conducted in accordance with the ethical standards of the 1964 Declaration of Helsinki and its amendments.

### Histological evaluation

All pathological features of the resected specimens or biopsies were evaluated at each institution. The pathological features (histological type, invasion depth, vascular invasion, and lymph-node metastasis) of the specimens resected using endoscopy or surgery were evaluated according to the JCCAC. The venous and lymphatic invasion was evaluated using hematoxylin and eosin staining. Elastic fiber staining (Victoria blue, Elastica van Gieson) and immunostaining (D2-40) were performed to confirm lymphovascular invasion at the discretion of pathologists as necessary in each institution.

### Investigated variables

The following clinicopathological variables were evaluated: age, sex, history of malignancy of other organs, family history of malignancy (parents, children, and brothers/sisters), history of Lynch syndrome, opportunity for diagnosis, presence of symptoms, anemia, vomiting, abdominal pain, bowel obstruction at diagnosis, treatment method, tumor location, tumor size, growth type, histological type, tumor invasion depth, presence of lymphatic invasion, venous invasion, lymph-node metastasis, tumor stage, and site of metastasis. The prognosis for each tumor stage was evaluated, along with the 5-year overall survival (OS) and 5-year disease-specific survival (DSS) rates. The OS rate was defined as the percentage of patients who remained alive for a certain period after diagnosis. The DSS rate was defined as the percentage of patients who did not die from PSBA within a certain period. Finally, the OS and DSS rates were compared based on the stage, tumor site, and presence/absence of symptoms at diagnosis.

### Statistical analysis

All data are shown as mean ± standard deviation, median, and percentage. The OS and DSS rates were calculated using the Kaplan–Meier method, and differences were compared using the log-rank test. When calculating the DSS rate, cases in which the cause of death was unknown were excluded. Cox regression analysis for DSS was performed to calculate the hazard ratios. Differences were considered statistically significant at *p*-value < 0.05. JMP statistical software version 16.0.0 (SAS Institute, Cary, NC, USA) was used for all statistical analyses.

## Results

### Clinicopathological features of enrolled patients

A total of 354 patients with 358 PSBA were enrolled in this study and their clinicopathological features are presented in Table 1. The median age of the enrolled patients was 67 (range 26–94) years, with 218 males (61.6%). The most common history of cancer was colorectal cancer (14.1%, 50 patients), followed by gastric cancer (7.6%, 27 patients), and the third was prostate cancer (2.8%, 10 patients). On the other hand, 28 patients (7.9%) had a history of multiple cancers. The rate of family history of malignancy was 18.6%. The proportion of patients diagnosed with Lynch syndrome was 5.4% (19 patients), and 15 of 19 patients had a history of colorectal cancer. Among the opportunities for diagnosis, 75.1% were examined for symptoms, 17.8% were incidental diagnoses (examination for other symptoms/diseases), and 7.1% were unknown. A total of 272 patients (76.8%) had various symptoms (anemia, vomiting, and abdominal pain) at diagnosis, and 121 patients (34.2%) had a bowel obstruction. The rates for anemia, vomiting, and abdominal pain were 40.1%, 31.6%, and 44.6%, respectively. Among the small-bowel endoscopies performed for diagnosis (61.9% of all patients), 12.1% were single-balloon endoscopies, 47.2% were double-balloon endoscopies, and 12.1% were capsule endoscopies (these data overlapped). Small-bowel endoscopy was performed more frequently in patients without obstruction (69.5%, 148/213) than in those with obstruction (53.7%, 65/121). Surgical resection was the most frequently performed treatment (291 patients, 82.2%), and 115 patients (32.5%) underwent chemotherapy after surgical resection. Of the 197 patients with clinical stages I–III, 131 patients (66.5%, 131/197) only underwent surgery, 56 patients (28.4%, 56/197) underwent chemotherapy after surgery, and two patients (1.0%, 2/197) only underwent chemotherapy. Similarly, among the 126 patients with clinical stages IV, 35 patients (27.8%, 35/126) only underwent surgery, 57 patients (45.2%, 57/126) underwent chemotherapy after surgery, and 25 patients (19.8%, 25/126) only underwent chemotherapy.

**Table 1:** Clinicopathological characteristics of enrolled patients with primary small bowel adenocarcinoma (*n* = 354)

Variables	
Age (years old)	
Median (range)	67 (26–94)
Sex	
Male	218 (61.6)
Female	136 (38.4)
History of malignancy of other organs *	
Colorectal cancer	50 (14.1)
Gastric cancer	27 (7.6)
Prostate cancer	10 (2.8)
Uterine cancer	6 (1.7)
Ovarian cancer	6 (1.7)
Bladder cancer	6 (1.7)
Lung cancer	5 (1.4)
Breast cancer	4 (1.1)
Duodenal cancer	3 (0.9)
Kidney cancer	3 (0.9)
Esophageal cancer	3 (0.9)
Others	11 (3.1)
Multiple cancers	28 (7.9)
No	220 (62.1)
Family history of malignancy (parents, children and brothers/sisters)	
Yes	66 (18.6)
No	182 (51.4)
Unknown	106 (30.0)
Lynch syndrome	
Yes	19 (5.4)
No	297 (83.9)
Unknown	38 (10.7)
Opportunity for diagnosis	
Examination for symptom	266 (75.1)
Incidental diagnosis (examination for other symptom/ disease)	63 (17.8)
Unknown	25 (7.1)
Symptom	
Yes	272 (76.8)
No	63 (17.8)
Unknown	19 (5.4)
Anemia	
Occult OGIB	99 (28.0)
Overt OGIB	43 (12.1)
No	172 (48.6)
Unknown	40 (11.3)
Vomiting	
Yes	112 (31.6)
No	211 (59.6)
Unknown	31 (8.8)
Abdominal pain	
Yes	158 (44.6)
No	167 (47.2)
Unknown	29 (8.2)
Bowel obstruction	
Yes	121 (34.2)
No	213 (60.2)
Unknown	20 (5.6)
Examinations for diagnosis	
Small bowel endoscopy*	219 (61.9)
Single-balloon endoscopy	43 (12.1)
Double-balloon endoscopy	166 (47.2)
Capsule endoscopy	43 (12.1)
Other examinations	135 (38.1)
Treatment	
Endoscopic resection	11 (3.1)
Open surgery	119 (33.6)
Open surgery and chemotherapy	85 (24.0)
Laparoscopic surgery	57 (16.1)
Laparoscopic surgery and chemotherapy	30 (8.5)
Chemotherapy	28 (7.9)
Palliative therapy	18 (5.1)
Unknown	6 (1.7)
Follow-up period (months, mean ± SD)	31.0 ± 30.8
Recurrence (Stage 0-III)	51/ 216 (23.6)
Disease specific death	106 (29.9)
	(%)

^*^The following data are overlapping

SD: standard deviation, OGIB: obscure gastrointestinal bleeding

### Clinicopathological features of enrolled PSBAs

The clinicopathological features of enrolled PSBAs are shown in Table 2. PSBA was most commonly located in the jejunum (66.2% of cases), and the average distance from the ligament of Treitz was 32.7 ± 36.9 cm. The average tumor size was 49.9 ± 27.9 mm, excluding 75 tumors with no data on size. Type 2 (54.2%) was the most common among the macroscopic types, followed by Type 3 (18.2%). The incidence of papillary adenocarcinoma and well/moderately differentiated tubular adenocarcinoma (differentiated carcinoma type) was 73.5%, whereas that of poorly differentiated adenocarcinoma, mucinous adenocarcinoma, signet ring cell carcinoma, and undifferentiated carcinoma (undifferentiated carcinoma type) was 21.7%. Tumor invasion depth was mostly T3 (29.3%) or T4 (50.6%). Lymph -node metastasis was observed in 116 of the 291 patients (39.8%) after surgical resection. The rates for clinical stages 0, I, II, III, and IV at the time of diagnosis were 5.4%, 2.5%, 27.1%, 26.0%, and 35.6%, respectively.

**Table 2:** Clinicopathological characteristics of enrolled primary small bowel adenocarcinoma (*n* = 358)

Variables	
Location	
Jejunum	237 (66.2)
Distance from Treitz’ ligament (cm, mean ± SD)	32.7 ± 36.9
Distance from pyloric ring (cm, mean ± SD)	59.2 ± 39.0
Unknown	128 (35.8)
Ileum	109 (30.4)
Distance from ileocecal valve (cm, mean ± SD)	38.3 ± 53.3
Unknown	62 (17.3)
Jejunum and ileum	6 (1.7)
Unknown	6 (1.7)
Size (mm)	
〜10	4 (1.1)
11–20	28 (7.8)
21–30	40 (11.2)
31–40	63 (17.6)
41–50	46 (12.8)
51–60	36 (10.1)
61–70	21 (5.8)
71–80	17 (4.7)
81–90	8 (2.2)
91–	20 (5.6)
–1/4 circumference	2 (0.6)
< 1/4〜1/2 circumference	7 (2.0)
< 1/2〜3/4 circumference	5 (1.4)
< 3/4〜entire circumference	35 (9.8)
Unknown	26 (7.3)
Macroscopic type	
0-Is	8 (2.2)
0-Isp	3 (0.8)
0-Ip	3 (0.8)
0-IIa	3 (0.8)
0-IIc	5 (1.4)
Type 1	32 (8.9)
Type 2	194 (54.2)
Type 3	65 (18.2)
Type 4	3 (0.8)
Type 5	12 (3.4)
Submucosal tumor type	9 (2.6)
Unknown	21 (5.9)
Histological type	
Papillary adenocarcinoma	9 (2.5)
Well differentiated tubular adenocarcinoma	108 (30.2)
Moderately differentiated tubular adenocarcinoma	146 (40.8)
Poorly differentiated tubular adenocarcinoma (solid type)	33 (9.2)
Poorly differentiated tubular adenocarcinoma (non-solid type)	13 (3.6)
Mucinous adenocarcinoma	20 (5.6)
Signet ring cell carcinoma	4 (1.1)
Undifferentiated carcinoma	8 (2.2)
Others	4 (1.1)
Unknown	13 (3.7)
Tumor invasion depth	
Tis	21 (5.8)
T1	5 (1.4)
T2	5 (1.4)
T3	105 (29.3)
T4	181 (50.6)
Unknown	41 (11.5)
Lymphatic invasion	
Positive	184 (51.4)
Negative	98 (27.3)
Unknown	76 (21.3)
Venous invasion	
Positive	193 (53.9)
Negative	87 (24.3)
Unknown	78 (21.8)
Lymph node metastasis after surgical resection	
Positive	116/ 291 (39.8)
Negative	127/ 291 (43.6)
Unknown	48/ 291 (16.6)
Clinical stage (for patients)	
0	19 (5.4)
I	9 (2.5)
II	96 (27.1)
III	92 (26.0)
IV	126 (35.6)
Unknown	12 (3.4)
Site of metastasis at first diagnosis (Stage IV)*	
Peritoneum	72/ 126 (57.1)
Liver	34/ 126 (27.0)
Lymph node	16/ 126 (12.7)
Lung	15/ 126 (11.9)
Bone	3/ 126 (2.4)
Brain	2/ 126 (1.6)
Spleen	2/ 126 (1.6)
Small intestine	2/ 126 (1.6)
Adrenal gland	2/ 126 (1.6)
Ovary	2/ 126 (1.6)
Others	4/ 126 (3.2)
Double site of metastases	21/ 126 (16.7)
Triple site of metastases	6/ 126 (4.8)
Unknown	10/ 126 (7.9)
	(%)

^*^The following data are overlapping

*SD* standard deviation

### Prognosis of enrolled patients with PSBA

**Fig. 2:**
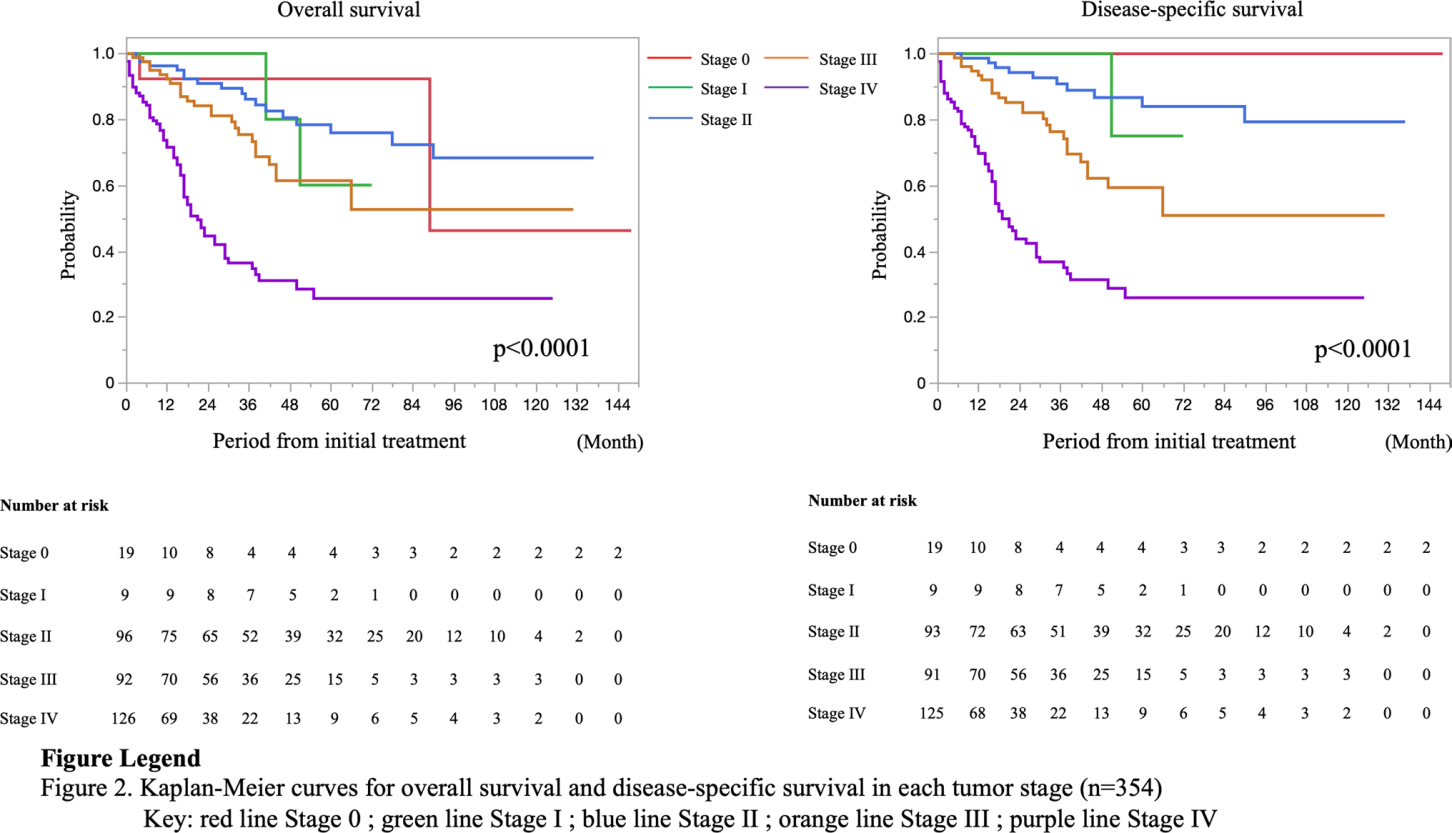
Prognosis of 354 patients with primary small bowel adenocarcinoma according to the clinical stage

**Fig. 3:**
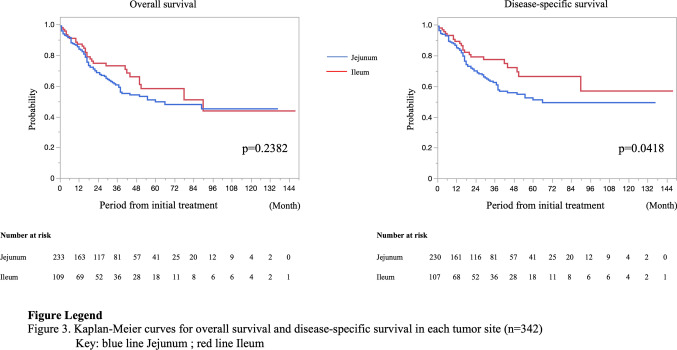
Prognosis of 342 patients with primary small bowel adenocarcinoma according to the tumor site

**Fig. 4:**
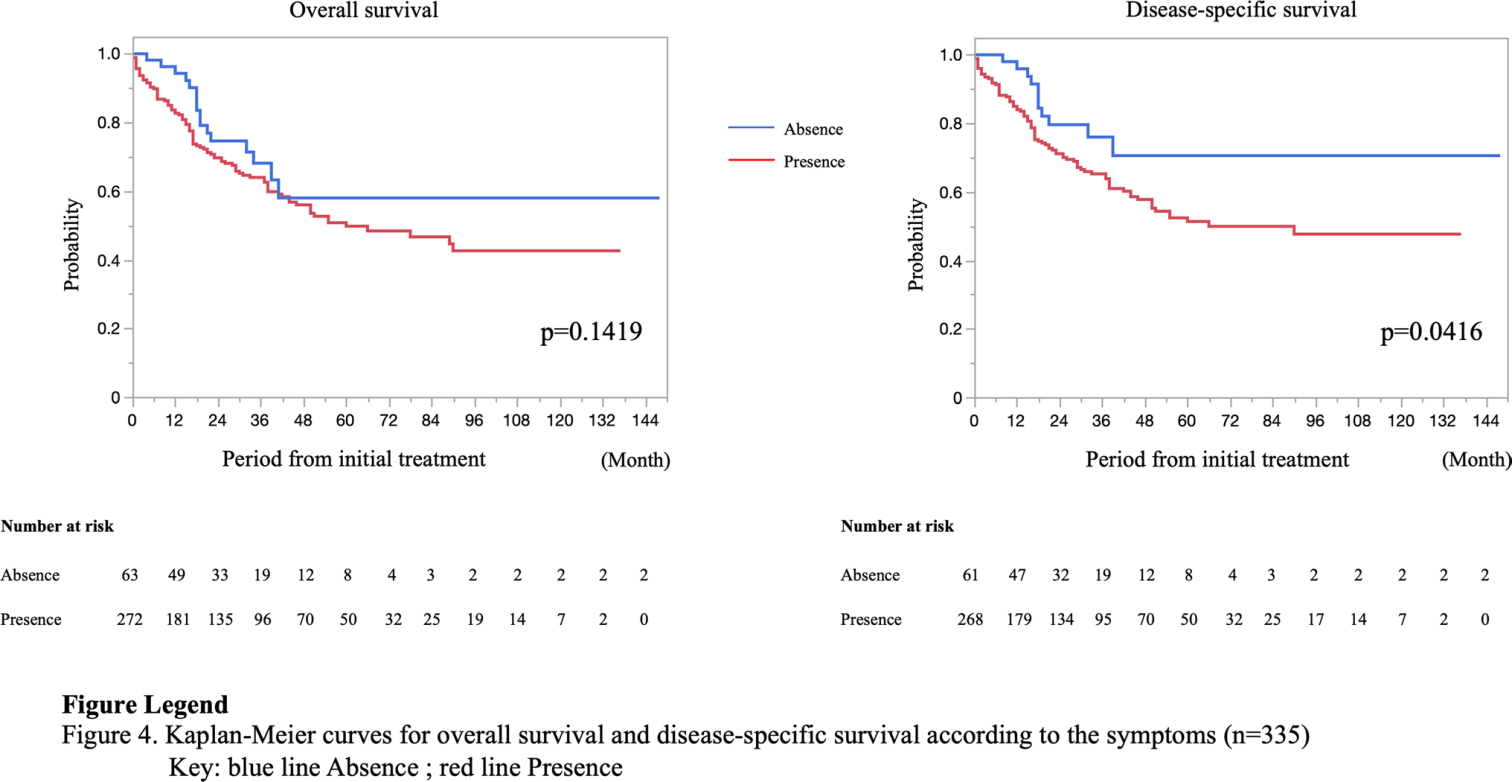
Prognosis of 335 patients with primary small bowel adenocarcinoma according to the presence or absence of symptoms

In the present study, the average follow-up period was 31.0 ± 30.8 months. There were 51 recurrences in 216 patients with stage 0–III PSBA. The recurrence rates were as follows: stage 0, 5.3% (1/19); stage I, 11.1% (1/9); stage II, 19.4% (18/96); and stage III, 33.7% (31/92). The most common site of recurrence and metastasis for stage I–III during the follow-up period was the peritoneum (13.2%), followed by the liver (6.6%), and the third was the lymph node (4.1%) (these data overlapped). Ten of nineteen patients with stage 0 underwent endoscopic resection, and one patient who underwent polypectomy had a local recurrence 2 months after treatment. A patient with stage I recurrence underwent surgical resection and was diagnosed with pT1pN0; lung metastasis was detected 39 months after the initial treatment. Similarly, the most common site of metastasis at the first diagnosis (stage IV) was the peritoneum (57.1%), followed by the liver (27.0%), and the third was the lymph node (12.7%). Moreover, 21 patients (16.7%) had double sites of metastases, and six patients (4.8%) had triple sites of metastases. The 5-year OS and 5-year DSS rates were 92.3% and 100% in stage 0, 60.0% and 75.0% in stage I, 75.9% and 84.1% in stage II, 61.4% and 59.3% in stage III, and 25.5% and 25.6% in stage IV, respectively (Fig. 2). The 5-year OS rate was analyzed after excluding 12 cases of unknown stage or unknown survival, and the 5-year DSS rate was analyzed after excluding five cases in which the cause of death was unknown. Moreover, the prognosis was compared according to the tumor site and the presence/absence of symptoms at diagnosis. The rates for clinical stages 0, I, II, III, and IV of the jejunum were, 2.7%, 0.4%, 27.7%, 27.2%, and 42.0%, respectively, and of the ileum were 12.1%, 7.5%, 28.0%, 26.2%, and 26.2%, respectively (*p* < 0.0001). Patients with the PSBA in the jejunum had a significantly lower 5-year DSS rate than those with the PSBA in the ileum (50.8% vs. 66.7%, *p* = 0.0418) (Fig. 3). The rates for clinical stages 0, I, II, III, and IV for patients with symptoms were 1.5%, 1.1%, 27.6%, 29.4%, and 40.4% and without symptoms were 18.0%, 9.8%, 27.9%, 14.8%, and 29.5%, respectively (*p* < 0.0001). The 5-year DSS rate in patients with symptoms at the initial diagnosis was significantly lower than in patients without symptoms (51.2% vs. 70.5%, *p* = 0.0416) (Fig. 4). Table 3 summarizes the results of the Cox regression analysis of DSS. Clinical stage was a significant predictor of DSS for patients with PSBC (*p* < 0.0001). On the other hand, age, sex, symptom, and tumor location were not significant predictors of DSS according to the multivariate analysis.

**Table 3:** Cox regression analysis for disease-specific survival in patients with primary small bowel adenocarcinoma (*n* = 354)

Variables		Univariate analysis			Multivariate analysis		
		HR	95% CI	*P*-value	HR	95% CI	*P*-value
Age (years)	< 65	1	0.77–1.66	0.5218	1	0.85–1.96	0.2390
	≥ 65	1.13			1.29		
Sex	Male	1	0.65–1.43	0.8613	1	0.71–1.68	0.6873
	Female	0.97			1.09		
Symptom	No	1	1.01–3.53	0.0465	1	0.97–3.97	0.0622
	Yes	1.89			1.96		
Tumor location	Ileum	1	1.01–2.59	0.0451	1	0.71–1.97	0.5204
	Jejunum	1.62			1.18		
Stage	0 and I	1			1		< 0.0001
	II	2.16	0.28–16.9	0.4626	1.21	0.15–10.1	
	III	6.43	0.87–47.6	0.0682	3.89	0.50–30.3	
	IV	20.6	2.85–148	0.0027	14.8	1.96–111	

*CI* confidence interval, *HR* hazard ratio

## Discussion

This is the first report to include large amounts of data from a multicenter study in Japan and examine the clinicopathological features and prognosis of PSBA in detail. Previously, it was difficult to detect lesions on the anal side of the ligament of Treitz. Moreover, endoscopic biopsy was difficult; therefore, surgery resection was often required to make a diagnosis [16, 17, 20]. Owing to recent advances in the field of small-bowel endoscopy, especially in diagnostic abilities and therapeutic techniques, the incidence of small bowel tumors initially diagnosed using biopsy and treated with endoscopic resection has increased [18, 19]. However, PSBA is still often detected at an advanced stage, with metastasis to other organs or peritoneal dissemination, because of its rarity [2–4]. Therefore, the risk factors for PSBA should be examined, and high-risk cases should be treated at an early stage. Crohn’s and celiac diseases have been reported as risk factors for PSBA [7, 8, 12, 13]. These diseases result in PSBA against a background of chronic inflammation; however, appropriate small intestinal surveillance methods and the duration of these diseases have not been established [21, 24]. Similarly, reportedly, hereditary diseases, such as familial adenomatous polyposis [9], Peutz-Jeghers syndrome [10], and Lynch syndrome [11, 14, 24], are also risk factors for PSBA. Lynch syndrome is a disease in which germline mutations in mismatch-repair genes (*MLH1, MSH2, MSH6, EPCAM, and PMS2*) predispose patients to the development of various tumors [24]. Because of the extremely low incidence of PSBA in the general population, the proportion of tumors associated with Lynch syndrome is relatively high, at approximately 4–8% among small intestinal cancers [11, 14]. In the present study, the proportion of patients diagnosed with Lynch syndrome was 5%, which was similar to that reported previously. However, this was only a report of diagnosed cases, and the actual incidence of Lynch syndrome may have been higher. Lynch syndrome is predisposed to colorectal cancer; in fact, 15 of the 50 patients in this study with a history of colorectal cancer had Lynch syndrome. Therefore, patients with a history of colorectal cancer may include undiagnosed cases of Lynch syndrome. Although the usefulness of small-bowel capsules for surveillance of Lynch syndrome has been reported [25], the surveillance intervals or which cases of Lynch syndrome are at a high risk for PSBA remain unknown.

Approximately 75% of all patients had symptoms, with the most common being abdominal pain, followed by vomiting. These symptoms are nonspecific and have been reported in the previous studies [7, 16, 17, 20, 26, 27]. Talamonti et al. [26] reported that patients usually had symptoms for a long time before diagnosis, with a mean duration of 10 months. Thus, if these symptoms persist for a long time, an examination of the small intestine should be performed.

Small-bowel endoscopy (single-balloon endoscopy [28], double-balloon endoscopy [18, 19], and capsule endoscopy [29]) is useful not only for the diagnosis but also for the treatment of small intestinal lesions. No study has reported the actual rate of small-bowel endoscopy as a diagnostic device for PSBA. In this study, small-bowel endoscopies were used in approximately 60% of the cases (with some cases overlapping), indicating their widespread use. Moreover, the PSBA site was measured by using small-bowel endoscopy, intraoperative findings, and surgical specimens in this study; the frequent PSBA sites were the proximal jejunum within 40 cm of the ligament of Treitz in the jejunum and the distal ileum within 40 cm of the ileocecal valve in the ileum.

Most previous reports on the prognosis of PSBA have included the duodenum, and some of these studies have revealed the duodenum itself as a poor prognostic factor [2, 30]. However, other studies reported that duodenum is a favorable prognostic factor [4, 31]. This may reflect differences in the number of cases per stage in each report and the inclusion of ampullary carcinoma [32]. Therefore, whether the duodenum is a prognostic factor for PSBA remains controversial. The prognosis of PSBA should be analyzed, excluding the duodenum; however, there are few reports on the prognosis. Amin et al. [23] reported that the 5-year OS rates in patients with PSBA from graph data were approximately 80% for stage I, 60% for stage II, 35% for stage III, and 10% for stage IV. Limited to stages I-III, the 5-year OS rate was 43–59% [2, 4, 20]. In contrast, the 5-year OS rate was < 10% in patients with stage IV disease [4, 20]. Based on the findings of the previous studies and those of our study, the tumor stage is the most important prognostic factor in PSBA [16, 20, 32]. In our study, the 5-year OS and DSS rates for stage I were lower than those for stage II because of the small number of patients in stage I and the fact that stage I patients included many older patients with comorbidities. The reported rates of PSBA stage were 3–11% for stage 0–I, 23–38% for stage II, 22–30% for stage III, and 32–41% for stage IV [2–4, 7, 8, 17, 20, 31]. The rate of each stage in our study was similar to that reported in the previous studies; however, the survival rate was higher. The first reason for this was the high rate of surgical resection (approximately 80%) in our study. Curative resection is the main treatment strategy for PSBA, and many surgical resections are performed at the localized stage [21, 32]. In actual practice, in patients with PSBA, surgical resection is often performed even at stage IV if there are obstructive symptoms or perforation findings [17, 27]. In such cases, resection of the primary tumor (radical or non-radical) or bypass surgery is performed, followed by chemotherapy. Furthermore, surgical resection may be performed even for resectable distant metastases. These resections may have increased the efficacy of systemic chemotherapy, because they reduced the tumor volume or improved patients’ activities of daily life. Second, the extent of lymph-node dissection was determined according to the JCCAC, which may have contributed to the prognosis. In fact, lymph-node metastasis was an unfavorable prognostic factor for localized PSBA [16, 26], and several reports indicated that the number of lymph-node dissections was a prognostic factor [3, 33]. Third, various new chemotherapeutic regimens have been developed in recent years. In addition to 5-fluorouracil, capecitabine, oxaliplatin, cisplatin, and irinotecan, which were commonly used for PSBA treatment [21, 32], bevacizumab, regorafenib, or anti-EGFR monoclonal antibodies can be used. Moreover, immune checkpoint inhibitors have been available since 2014 in Japan and are expected to be effective for PSBA in the future. In particular, Lynch syndrome is correlated with mismatch-repair deficiency, which is a good indication for the use of immune checkpoint inhibitors [34]. The peritoneum was the most common site of PSBA metastasis, followed by the liver and lungs, and the same outcome was observed in this study [7, 27]. The rate of peritoneal metastasis was approximately 30–50% in PSBA [7, 27], which could cause obstructive symptoms and was a major factor that made curative surgical resection impossible. The small intestine has a thinner wall than the other gastrointestinal tracts and is presumably more prone to peritoneal dissemination.

The Kaplan–Meier method revealed that the presence of symptoms at the initial diagnosis and the tumor location in jejunum were associated with significantly worse prognosis. However, according to the multivariate analyses with Cox-hazard model, clinical stage was only significant predictor of DSS for patients with PSBC. Similar to the present study, several studies have reported that the presence of symptoms at the initial diagnosis is a poor prognostic factor [7, 27]. Since there were no specific symptoms of PSBA, the disease may have already progressed when symptoms, such as vomiting, appeared. However, Sakae et al. [7] reported that the presence of symptoms at diagnosis was an independent prognostic factor for the tumor stage. Tian et al. [27] also reported that the multivariate predictors of poor prognosis were intestinal obstruction or perforation at first diagnosis. Based on these reports, symptomatic PSBA itself may exhibit poor oncological behavior. Further research, including genetic analysis, is needed to confirm this hypothesis. Moreover, the DSS rate was significantly lower for the jejunum than for the ileum in our study. This result differed from that of previous reports in that the ileum was a poor prognostic factor [4]. This may be because the rate of stage IV tumors was higher in the jejunum than in the ileum in our study.

This study had some limitations. First, this study had an inevitable selection bias, because the data were collected retrospectively from relatively high-volume centers; a prospective multicenter study should be conducted to optimize the treatment for PSBA, a rare disease. Second, not all cases were genetically screened and may potentially include a greater number of patients with Lynch syndrome. Third, only the surgical technique was examined, and it is unknown whether curative surgery was performed. Finally, because there were no PSBA guidelines, treatment decisions were made at the discretion of each institution (most cases were treated in accordance with the JCCAC and JSCCR guidelines). Therefore, there is an urgent need to establish guidelines for PSBAs.

In conclusion, we identified the characteristics and prognoses of patients with PSBA in a large number of cases. To improve the PSBA prognosis, high-risk patients, such as those with Lynch syndrome, should be identified and screened, and PSBA should be detected and treated in the early stages before symptoms appear.
